# Microbial-assisted soil chromium immobilization through zinc and iron-enriched rice husk biochar

**DOI:** 10.3389/fmicb.2022.990329

**Published:** 2022-09-12

**Authors:** Masooma Batool, Shafeeq ur Rahman, Muhammad Ali, Faisal Nadeem, Muhammad Nadeem Ashraf, Muhammad Harris, Zhenjie Du, Waqas-ud-Din Khan

**Affiliations:** ^1^Sustainable Development Study Centre, Government College University, Lahore, Pakistan; ^2^School of Environment and Civil Engineering, Dongguan University of Technology, Dongguan, China; ^3^MOE Laboratory for Earth Surface Processes, College of Urban and Environmental Sciences, Peking University, Beijing, China; ^4^Department of Soil Science, University of the Punjab, Lahore, Pakistan; ^5^Institute of Soil & Environmental Sciences, University of Agriculture, Faisalabad, Pakistan; ^6^Department of Environmental Sciences, University of Lahore, Lahore, Pakistan; ^7^Farmland Irrigation Research Institute, Chinese Academy of Agricultural Sciences, Xinxiang, China; ^8^Water Environment Factor Risk Assessment Laboratory of Agricultural Products Quality and Safety, Ministry of Agriculture and Rural Affairs, Xinxiang, China

**Keywords:** doped biochar, wastewater (WW), fungi and bacteria, phylogenetic analysis, heat map analysis

## Abstract

Soil chromium toxicity usually caused by the tannery effluent compromises the environment and causes serious health hazards. The microbial role in strengthening biochar for its soil chromium immobilization remains largely unknown. Hence, this study evaluated the effectiveness of zinc and iron-enriched rice husk biochar (ZnBC and FeBC) with microbial combinations to facilitate the chromium immobilization in sandy loam soil. We performed morphological and molecular characterization of fungal [*Trichoderma harzianum* (F1), *Trichoderma viride* (F2)] and bacterial [*Pseudomonas fluorescence* (B1), *Bacillus subtilis* (B2)] species before their application as soil ameliorants. There were twenty-five treatments having ZnBC and FeBC @ 1.5 and 3% inoculated with bacterial and fungal isolates parallel to wastewater in triplicates. The soil analyses were conducted in three intervals each after 20, 30, and 40 days. The combination of FeBC 3%+F2 reduced the soil DTPA-extractable chromium by 96.8% after 40 days of incubation (DAI) relative to wastewater. Similarly, 92.81% reduction in chromium concentration was achieved through ZnBC 3%+B1 after 40 DAI compared to wastewater. Under the respective treatments, soil Cr(VI) retention trend increased with time such as 40 > 30 > 20 DAI. Langmuir adsorption isotherm verified the highest chromium adsorption capacity (41.6 mg g^−1^) with FeBC 3% at 40 DAI. Likewise, principal component analysis (PCA) and heat map disclosed electrical conductivity-chromium positive, while cation exchange capacity-chromium and pH-organic matter negative correlations. PCA suggested the ZnBC-bacterial while FeBC-fungal combinations as effective Cr(VI) immobilizers with >70% data variance at 40 DAI. Overall, the study showed that microbes + ZnBC/FeBC resulted in low pH, high OM, and CEC, which ultimately played a role in maximum Cr(VI) adsorption from wastewater applied to the soil. The study also revealed the interrelation and alternations in soil dynamics with pollution control treatments. Based on primitive soil characteristics such as soil metal concentration, its acidity, and alkalinity, the selection criteria can be set for treatments application to regulate the soil properties. Additionally, FeBC with *Trichoderma viride* should be tested on the field scale to remediate the Cr(VI) toxicity.

## Graphical Abstract

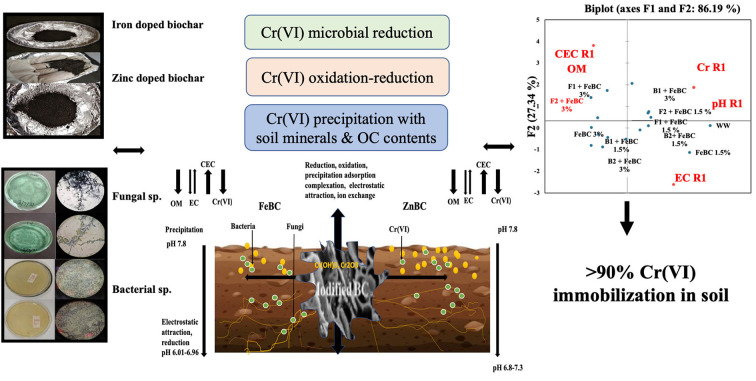

## Introduction

Heavy metals such as chromium Cr(VI), arsenic (As), and lead (Pb) are generally released from processing and manufacturing industries (Wang et al., 2020). Chromium exists in natural environment as H_2_CrO_4_ at pH <1, ${\text{HCrO}}_{4}^{-}$ at pH <6, and CrO_4_^2−^ at pH > 6.0 (Xia et al., 2019). Chromium is highly toxic and mutagenic due to its oxidization characteristics to the intracellular components of living organisms (Wei et al., 2022). Chromium [Cr(VI)] has 1,000 times more cellular toxicity potential compared to Cr(III) (Shahid et al., 2017). The Cr(VI) threatens the threshold limits of land and aquatic systems due to its high stability, persistence, and solubility nature (Chen et al., 2015). It is translocated from the soil to the plant body through sulfate and phosphate transporters (Ao et al., 2022). In plants, Cr(VI) damages protein structure and minerals (K, Fe, Ca, Mn, and Mg) balance (Srivastava et al., 2021). The Cr(VI) metal has a high capacity of bioaccumulation in the human body through the food chain (Azeez et al., 2021). Ahmad et al. (2012) also mentioned Cr(VI) induced cytotoxicity in human. For developing countries such as Pakistan, inadequate investment and advanced technology to treat concentrated industrial effluents always remained serious challenges (Abriz and Golezani, 2021). Some conventional metal removal methods such as the use of ferric salts, red mud, and inorganic clays have previously been reported; however, the application of inorganic materials leads to the formation of stable metal compounds having less mobility and accessibility to plants (Choo et al., 2020). Nevertheless, associated costs, production of secondary pollutants, and minimal metal removal rate remained the major constraints (Wang et al., 2020).

Biochar (BC) is a black carbonaceous compound produced from bio-wastes under anoxic conditions (Haider et al., 2021). Accessibility and variety of raw materials for biochar manufacturing make it an ideal adsorbent and an inexpensive soil fertility booster material (Dai et al., 2018). Biochar characteristics depend on the type of organic raw material, BC surface area, porosity, and magnetism [metal cations (Fe, Zn, etc.) enriched form] properties (Zhang et al., 2020). Rice husk (550–650°C) BC has a honeycomb-like structure with a low atomic H/C O/C ratio and volatile matter which contributes to its soil ameliorant properties (Pariyar et al., 2019). Through metal doping mechanisms, BC chemistry, reactivity, surface energy, and complex stability can be enhanced suitably to bring multiple grades of interaction among BC and soil components (Liu et al., 2019b; Moradi et al., 2019). In a study, wheat straw Fe-enriched BC (600°C) was added to Cr(VI)-laden soil (Lyu et al., 2018). At acidic pH, iron-sulfide (FeS) disassociated with Fe^2+^ and S^2−^ ions, and Fe(II) as a natural reductant reduced Cr(VI) to Cr(III) with a subsequent increase in Fe–Cr co-precipitates [(Fe, Cr) (OH)_3_]. The analysis on X-ray photoelectron spectroscopy (XPS) showed the disappearance of Fe°, and the appearance of Cr_2_O_3_ and Fe_2_O_3_ peaks confirmed Cr(VI) reduction by Fe° released electrons. In the same context, Li et al. (2018) showed that granular-activated carbonaceous BC enriched with zinc oxide (ZnO) resulted in a 5 times higher metal (Pb^2+^) removal rate compared to activated BC only. Magnetic BC (Fe/Zn enriched) produced (400°C) from rice husk expressed ~10 times higher Pb^2+^ adsorption capacity than simple BC (Seleiman et al., 2020).

The acidification-based enrichment process makes BC a soil pH-reducing agent (Moradi et al., 2019). The chromium oxide species such as negatively charged chromate ions (CrO^2−^; ${\text{CrO}}_{4}^{2-}$) are readily adsorbed at low pH (Zhang et al., 2018a). Contrarily, at high pH levels, OH^−^ species and Cr(VI) anionic species competitively interact for positively charged compounds (Gomes, 2016). The soil physicochemical properties such as organic matter (OM), pH, and electrical conductivity (EC) strongly influence the heavy metals immobilization by ion exchange, surface coagulation, sorption, chelation, and precipitation mechanisms; however, the bonding nature varies with metal characteristics (Saffari et al., 2016). Similarly, biologically modified systems through metabolism or bioaccumulation pathways contribute to metal adsorption and detoxification. Microbial phospholipid fatty acid (PLFA) studies revealed that bacteria and fungi vary in their soil OM degradation patterns through which their pollution remediation potential can be determined (Xu et al., 2018). The endophytes such as *Trichoderma* sp. develop mutual chemistry with soil dwellers (other bacterial and fungi) and release metabolites facilitating metal detoxification. Fungal mycelia usually excrete glomalin protein, which reduces the metal bioavailability (Shakya et al., 2016). Furthermore, fungal hyphae, spores, and vesicles work as exclusion barriers (Khan et al., 2020). Bacteria generally reduce the metal content by their metabolic pathways and primarily enhance the soil fertility and organic constituents' bioavailability through OM degradation (Hashem et al., 2019). Naggar et al. (2020) reported *Pseudomonas* sp. (*alcaliphila* NEWG strain) use for Cr(VI) removal (96.33%) from aqueous solution (pH 7) within 48 h of the incubation period. He et al. (2020) combined *ureolytic* bacteria with *P. fluorescence* for remediation of polluted soils and achieved 83% removal of Pb and cadmium (Cd) metals. Heavy metals accumulation by *P. fluorescenc*e appeared to be highly pH-dependent as it showed efficient metal removal at acidic pH (Lopez et al., 2000). Alotaibi et al. (2021) referred to the chromate reductase (ChrR) enzyme-producing Cr-resistant *Bacillus* sp. to reduce toxic Cr(VI) to Cr(III) with a 95% efficiency level. In another study, *B. subtilis* mediated ~96% Cd and 65% Cr pollution in edible radish parts relative to control (Wang et al., 2014). *B. subtilis* improved the antioxidant enzymes production in the Alfalfa plant and enhanced the nutrient cycling in the amended soil. The same species enhanced 29.4% alfalfa growth, whereas 139% Cd metal immobilization was observed in treated soil relative to control (Li et al., 2021). These microbial species are benign and have never been reported as pathogenic organisms (Quintieri et al., 2020; Su et al., 2020).

Although various studies mentioned the BC–microbial association in removing metal toxicity from different mediums such as aqueous solutions and soil (Han et al., 2016; Huang et al., 2017; Khan et al., 2020), however, few studies are available on enriched BC–microbial associations in remediation of tannery wastewater (WW) impacted soil (Batool et al., 2022; Meki et al., 2022). Based on microbial strains efficacy, bacterial species including *P. fluorescence* (B1) and *B. subtilis* (B2), and fungal sp. of genus Trichoderma including *T. harzianum* (F1) and *T. viride* (F2) were exclusively selected for the proposed study.

We hypothesized that the FeBC and ZnBC in combination with fungal and bacterial isolates could remediate soil Cr(VI) pollution. Primarily, the research was conducted to evaluate the effects of Cr(VI) polluted WW on soil physicochemical properties in control and how microbial–BC application improved these properties at different intervals. Similarly, the microbial–BC role in soil Cr(VI) removal was assessed through principal component analysis–heat map and Cr(VI) adsorption kinetics.

## Materials and methods

### Wastewater collection for soil spiking

The WW effluent was collected from the Siddique leather works (Pvt.) industry, Lahore (latitude: 31°37'48.05“N; longitude: 74°12'59.94”E), Pakistan. The composite WW samples were collected during November 2021 and preserved at −4°C until the experimentation. Before application to soil, WW was filtered through the filter paper (Whatman no. 42) to remove the suspended particles. Then, Cr(VI) concentration was analyzed through a multi-sequential Atomic Absorption Spectrophotometer (Thermo Scientific iCE 3000 AAS, USA) by following Syuhadah's et al. (2015) guidelines. The Cr(VI) metal concentration in WW was 523.18 ppm considered for soil spiking.

### Morphological and molecular characterization of microbial isolates

*T. harzianum* (F1) (accession code: FCBP-SF-1277), T. *viride* (F2) (accession code: FCBP-SF-644), *P. fluorescence* (B1) (accession code: FCBP-SB-0188), and *B. subtilis* (B2) (accession code: FCBP-SB-0189) cultures were acquired from the First Fungal Culture Bank of Pakistan (FCBP), University of the Punjab, Lahore.

Fungal species (F1, F2) were re-inoculated and maintained on Malt extract agar (10 g Malt extract, 10 g agar, 500 ml distilled water). The pH of the medium was adjusted to 6.5 with sodium hydroxide (NaOH). Fungal cultures were incubated at 26–28°C for 7 days (Baral et al., 2018). The purified culture morphological characterization was made by colony morphology and microscopic studies (**Table 2**; Sempere and Santamarina, 2011; Lagree et al., 2018). Finally, the pure cultures were stored at −4°C.

Fungal DNA was isolated by following the optimized Cetyl trimethylammonium bromide (CTAB) method (Doyle and Doyle, 1987) confirmed in gel electrophoresis (Inglis et al., 2018). PCR amplification was done with universal primers ITS 1 (5′TCCGTAGGTGAACCTGCGG3′) and ITS 4 (5′TCCTCCGCTTATTGATATGC3′). PCR-amplified product was sent for sequencing to Genetix: Gene Insight Experts Laboratory in Lahore (Gulberg-3), Pakistan. Through Sanger technology, obtained sequences were analyzed through BLAST (Basic Local Alignment Search Tool), and a phylogenetic tree was developed by MEGA-X software with the reference sequences from NCBI database to compare their genetic variation (**Figure 2**).

Bacterial cultures were re-inoculated on Lauria Bertin agar (Tryptone 5 g, yeast extract 2.5 g, NaCl 5 g, and agar 6 g in 500 ml d.H_2_O) medium (Loutfi et al., 2020). The plates were incubated at 34°C for 24 h. Morphological characterization of bacterial isolates was done by gram staining test, UV light test, colony morphology, and microscopic studies at 100X (Smith and Hussey, 2005; Moyes et al., 2009). Purified bacterial cultures were stored in 20% glycerol solution at −4°C (Howard, 1956). DNA of bacterial isolates was extracted by the phenol-chloroform method by following an optimized DNA extraction protocol (Holben, 1994). To confirm extracted DNA presence, gel electrophoresis (1% agarose gel with 1 X TAE buffer), PCR amplification (Supplementary Figure 1), and PCR product gel electrophoresis steps were followed (Riffiani et al., 2015). PCR amplification was performed by 16S rRNA primers. The amplified product was gel sequenced, and result sequences after BLAST analysis were processed for alignment and phylogenetic analysis by MEGA-X software.

The freshly grown fungal culture plates were added with 200 ml of d.H_2_O, and spores-enriched water was collected for pouring into treatment cups as 1 ml per 10 g of soil (David and Davidson, 2014). However, the bacterial broth cultures were prepared for direct application as 1 ml per 10 g of soil (Lederberg and Lederberg, 1952).

### Biochar preparation and characterization

Rice husk (oven-dried at 80°C for 24 h) grounded samples were pyrolyzed in a furnace (TF-1200X; Hefei Ke Jing Materials Technology Co. Ltd, China) at 550°C with 10°C/min heat rate (Dad et al., 2020). Then, the pyrolyzed material (BC product) was passed through a 2-mm sieve. For Fe and Zn enrichment on BC, the procedure was adopted as given in our following manuscripts (Dad et al., 2020; Taqdees et al., 2022). The physicochemical properties of enriched rice husk BC (ZnBC and FeBC) are listed in Table 1.

**Table 1:** Soil and biochar physicochemical properties (Mean ± S.D).

**Materials characterization**	**Type**	**Pyrolysis temperature**	**Texture**	**pH**	**EC (dS/m)**	**CEC [cmol (+)/Kg]**	**OM%**	**Cr mg kg^−1^**
Bhal soil	Loamy sand	–	Sand 75% Loam 18% Clay 7%	7.80 ± 0.3	0.8 ± 0.01	4.21 ± 0.4	0.8 ± 0.1	0.02 ± 0.001
Enriched-Biochar	ZnBC	550°C/min	–	4.7 ± 0.4	0.36 ± 0.02	14.9 ± 0.9	3.5 ± 0.6	–
	FeBC	10°C/min	–	2.67 ± 0.1	0.1134 ± 0.001	19.9 ± 1.2	8 ± 0.99	–

### Soil sample characterization

The soil used in this experiment was purchased from Malik nursery, Lahore, Pakistan. The physicochemical properties of the soil used in this experiment are given in Table 1. Soil texture was determined using the hydrometer method as described by Gee and Or (2002). Soil cation exchange capacity (CEC) was checked by the ammonium acetate extraction method (Ellen et al., 2001). For soil Cr(VI) metal content analyses, the diethylenetriamine penta-acetic acid (DTPA) metal-extraction method was followed (Lindsay and Norvell, 1978). Soil OM contents were quantified by the acid digestion (Walkley-black method) method (Nelson and Sommers, 1996). Soil pH and EC were checked with pH meter (smart CHEM-LAB Laboratory Analyser, VWR International Pty Ltd., Australia) (Rhoades, 1996) and EC meter (San ++, Skalar Analytical B.V., the Netherlands) (Hesse, 1971).

### Experimental setup and design

The research elements (Zn/Fe doped BC, microbial formulations, soil, and WW) were prepared as described in the above sections for pot (height: 15.2 cm; width 9.5 cm (circumference) and bottom 6.2 cm) fil before incubation trial. ZnBC and FeBC were measured as 1.5 and 3% rates with 60 g of Bhal soil as per pot. The 25 treatments such as WW (control), B1 (*P. fluorescence*), B2 (*B. subtilis*), F1 (*T. harzianum*), F2 (*T. viride*), ZnBC1.5%, ZnBC 3%, FeBC1.5%, FeBC3%, B1 + ZnBC1.5%, B1 + ZnBC3%, B1 + FeBC1.5%, B1 + FeBC3%, B2 + ZnBC1.5%, B2 + ZnBC3%, B2 + FeBC1.5%, B2 + FeBC3%, F1 + ZnBC1.5%, F1 + ZnBC3%, F1 + FeBC1.5%, F1 + FeBC3%, F2 + ZnBC 1.5%, F2 + ZnBC 3%, F2 + FeBC1.5%, and F2 + FeBC 3% were applied in pots (3 replicates each) for 3 assessment periods (20, 30, and 40 days). All the treatments were mixed under sterilized conditions to avoid spore contamination. Finally, WW (100% conc.; 40 ml) was applied to all the pots. Plastic pots were placed in the growth chamber at Sustainable Agriculture Laboratory, Sustainable Development Study Center (SDSC) Government College University, Lahore, under the uniform conditions (such as average temperature 25–30°C and humidity <20–40%).

### Soil post-harvest physicochemical parameter analyses

Soil samples were harvested after 20, 30, and 40 days of incubation (DAI). Each time, soil Cr(VI) concentration (Lindsay and Norvell, 1978; **Figure 4**), pH (Rhoades, 1996; **Figure 5**), OM content (Nelson and Sommers, 1996; **Figure 6**), EC (Hesse, 1971; **Figure 7**), and CEC (Richards, 1954; FAO, 1990; **Figure 8**) were determined. Similarly, the confirmation of viable fungal and bacterial spores in soil samples was performed by adopting the same procedures for re-isolation and characterization of the microbes as discussed in Section Morphological and Molecular Characterization of Microbial Isolates (**Figure 3**).

### Isotherm kinetic models

The Cr(VI) concentration adsorbed by adsorbents (unit mass) such as ZnBC and FeBC in soil was determined by the concentration difference (Manzoor et al., 2013; Shah et al., 2016). The mathematical expression was as follows:


$$\begin{array}{l} q=\left(Cf-Ci\right)\times \left(V/1000\right)/m \end{array}$$

Ci: Cr(VI) initial concentration (mg kg^−1^); Cf: Cr(VI) final concentration (mg kg^−1^); V: extractant volume (ml); m: adsorbent mass (g).

To calculate the percentage (%) metal removal, the control value was taken as an initial metal concentration (Ci) relative to treatments added as follows:


$$\begin{array}{l} C=Ci-Cf/Ci\times 100\; \end{array}$$

Langmuir and Freundlich's adsorption isotherm models were applied on Cr(VI) metal adsorption data (triplicate sets mean values) to distinguish ZnBC and FeBC adsorption capacities over time (Manzoor et al., 2013). The Langmuir linear equation (Equation 1) and Freundlich isotherm log equation (Equation 2) mathematical expressions are given


(1) $$\begin{array}{r} Ce/qe\;=\;1/qmaxK_{\text{l}}\;+\;Ce/qmax \end{array}$$

(2) $$\begin{array}{r} Log\;qe\;=\;1/n\;\left(Log\;Cf\right)\;+\;Log\;Kf \end{array}$$

Qe: Metal ions [Cr(VI)] sorbed (g kg^−1^); K_l_: Langmuir constant; Kf and 1/n: Freundlich constants.

### Statistical and co-linearity analyses (PCA, heat map)

The data were statistically analyzed by completely randomized design (CRD) using Sigma-plot (14.5 version) and Statistix@ 8.1 software (Tallhase software package, USA) to segregate the treatments based on the least significant difference (LSD) test (Liu et al., 2020). Further to evaluate the co-linearity and co-association of variables, principal component analysis (PCA–Pearson correlation) was applied to data sets (organized as ZnBC and FeBC treatments) with mean values for 20, 30, and 40 DAI by XLSTAT 2021 tool (Addinsoft-New York, USA). Finally, for all the treatment variables, a heat map was generated through the “*Metabo-analyst*” statistic analyses online website (https://www.metaboanalyst.ca/) to visually distinguish interval-wise changes in soil properties under metal stress and treatment additions individually.

## Results

**Figure 1:**
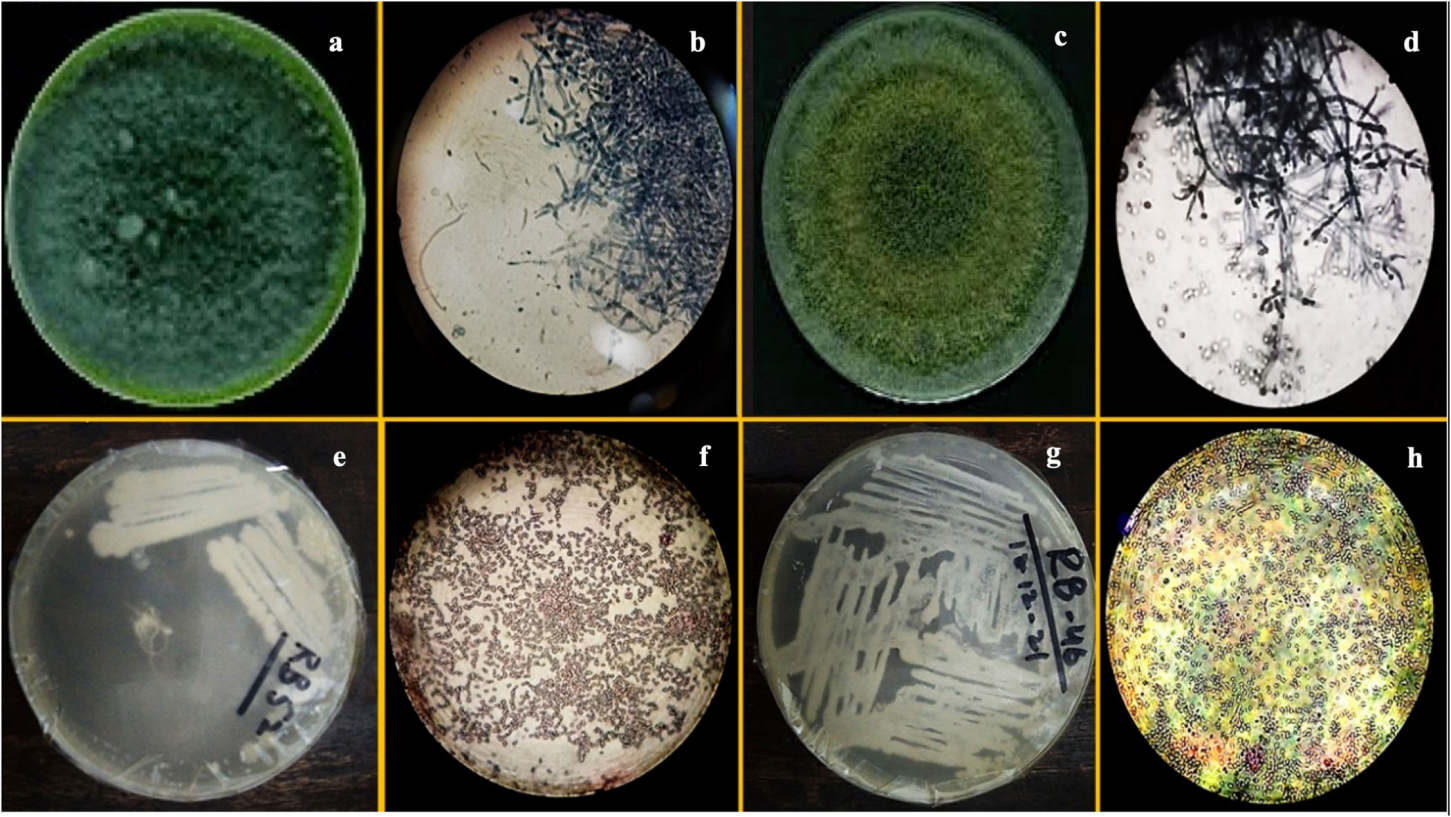
Images of pure culture T. *harzianum* **(a)** inoculated plate **(b)** microscopic study, pure culture T. *Viride* **(c)** inoculated plate **(d)** microscopic study; images of pure culture *Azotobacter nigricans* sp. **(e)** inoculated plate **(f)** microscopic study and pure cultures *Trichococcus* sp. **(g)** inoculated plate **(h)** microscopic study.

F1 had one concentric ring that appeared outward from the inoculum point and green conidia (with dense middle) were spread over the growth media having a compact mycelial growth pattern (Figure 1). In contradiction, F2 showed regular concentric growth rings and fluffy mycelium. Phialide (F1: 4 × 2.6 μm; F2: 3 × 3.2 μm) were hook-shaped in both sp. such as *Trichoderma* sp. (F1, F2) granular mycelial hyphae were aseptate, and conidiophores showed tuft of fertile conidia [F1: globose to subglobose (2.8 × 2.7 μm); F2: globose to ellipsoidal (3.2 × 2.7 μm)] attached at conidiophores (Figure 1). F1 conidiophores (4.5–7.0 × 2.7–2.5 μm) were frequently branched contrary to F2 which had irregularly branched conidiophore (5.0–6.5 × 1.7–2.7 μm) structures (Table 2).

**Table 2:** Selected microbial species characterization.

**Coding**	**Name**	**Culture accession no**.	**Color**	**Shape**	**Gram test**	**Spore**	**UV test**
**Bacteria species**							
B1	*P. fluorescence*	FCBP-SB-0188	Off-white	Rod	–	–	Bright
B2	*B. subtilis*	FCBP-SB-0189	Off-white	Rod	+	+	Bright
**Coding**	**Name**	**Culture accession no**.	**Morphology**	**Micro-metery**			
**Fungal species**							
F1	*T. harzianum*	FCBP-SF-1277	Rings: Multiple concentric rings and green (dense) middle Colony color: Green Colony texture: Compact, granular Hyphae: Septate Conidia shape: Globose to sub-globose Conidia color: Green Conidiophore: Frequently branched Chlamydospore formation: Infrequent, internal and terminal	Conidia size: 2.8 ×2.7 μm Phialide size: 4 ×2.6 μm Conidiophore size: 4.5 ~ 7.0 ×2.7 ~ 2.5 μm			
F2	*T. viride*	FCBP-SF-644	Rings: Enclosed rings with green conidia throughout or mostly outward Colony color: Green Colony texture: Fluffy, granular Hyphae: Septate Conidia shape: Globose to ellipsoidal Conidia color: Green Conidiophore: long, infrequently and irregularly branched, verticillate Chlamydospore formation: Terminal and intercalary	Conidia size: 3.2 ×2.7 μm Phialide size: 3 ×3.2 μm Conidiophore size: 5.0 ~ 6.5 ×1.7 ~ 2.7 μm			

The morphological characterization of bacterial isolates revealed rod-shaped B1 cells, which existed individually as well as in chain-like arrangements. Likewise, B2 cells were also rod-shaped but only appeared as single cells rather than chain or clustered forms (Figure 1, Table 2). Both sp. showed different gram staining projects such as B1 were gram-negative while B2 remained gram-positive as it retained crystal violet dye. In spore testing results, B1 was spore-positive while B2 persisted as negative. Both bacterial isolates were off-white. Similarly, both isolates appeared bright under UV radiations (Table 2).

**Figure 2:**
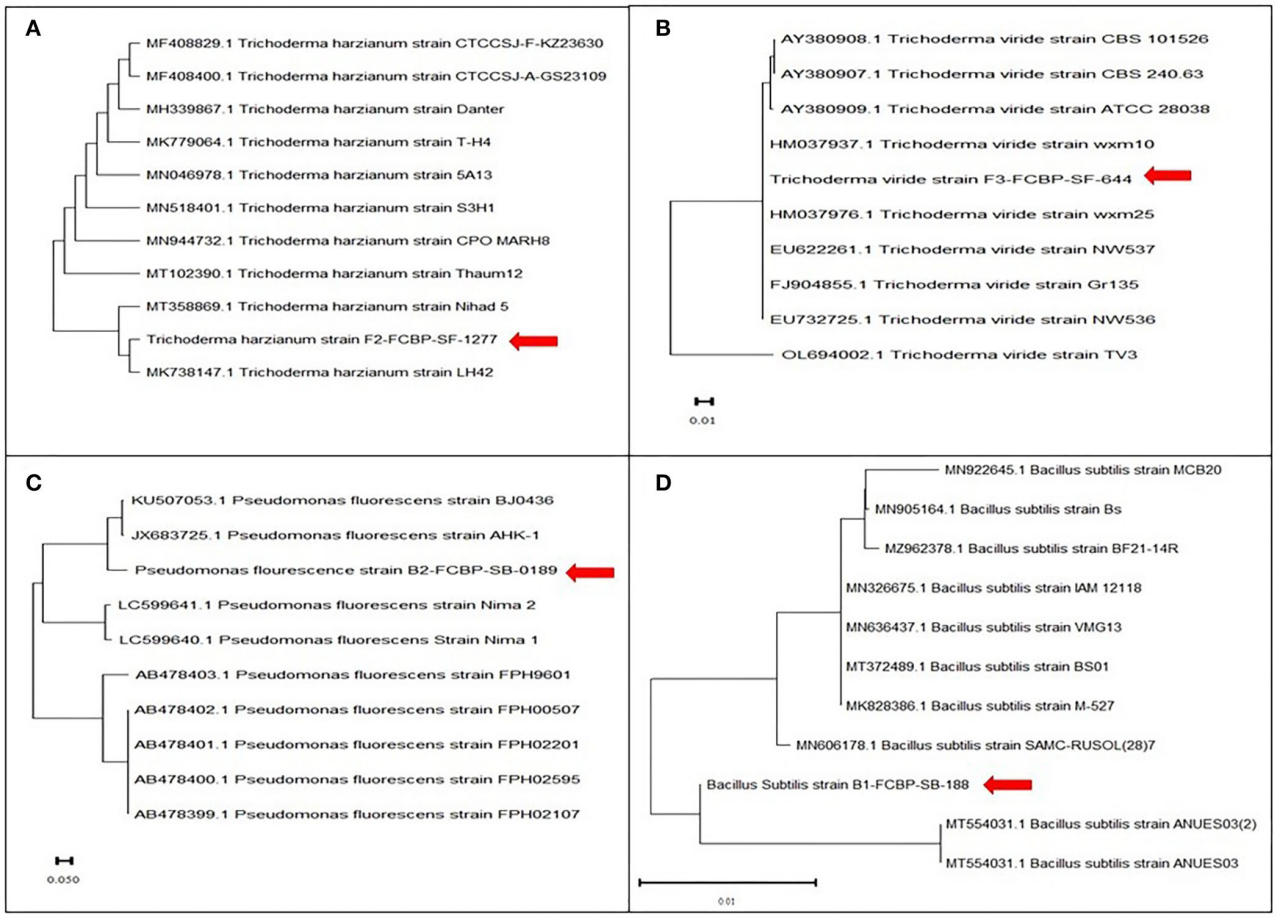
Phylogenetic dendogram of **(A)** *T. Harzianum*, **(B)** *T. viride* (sequenced with ITS1 and ITS4 regions), **(C)** *P. fluorescens*, and **(D)** *B. subtilis (*sequenced with 16S ribosomal RNA gene) for identification using sanger sequencing. Phylogenetic tree was constructed using the maximum likelihood method in MEGA version 10 following Tamura and Nei model. Bootstrap value was kept 1,000 replicates.

Extracted DNA bands from pure fungal (F1, F2) cultures were visible over agarose gel (DNA marker size 100 bp) before and after PCR amplification (Supplementary Figure 1). Molecular evolutionary genetics analysis of Trichoderma isolates revealed that F1 was genetically closer (>99.86% likelihood) to F2 as confirmed by NCBI reference sequences analyses. Phylogenetic dendrograms of *Trichoderma* sp. have been presented (Figure 2). Similarly, extracted DNA bands from pure bacteria (B1; B2) cultures appeared by gel electrophoresis (DNA marker size 100 bp) before and after PCR amplification were seen over agarose gel under UV luminescence (Supplementary Figure 1). Molecular evolutionary genetics analysis of B1 with its reference sequences from NCBI confirmed that the isolate had maximum genetic likelihood (92.04%) with an isolate of the same species from China and submitted to NCBI with an accession number KU507053, and 99.93% with another Chinese isolate with accession number JX683725. Likewise, B2 molecular evolutionary genetics analysis with its reference sequences from NCBI has confirmed isolate 99.86% genetic similarity with an isolate of the same species from India and submitted to NCBI with an accession number MK828386. Phylogenetic dendrograms of B1 and B2 have been given in Figure 2.

**Figure 3:**
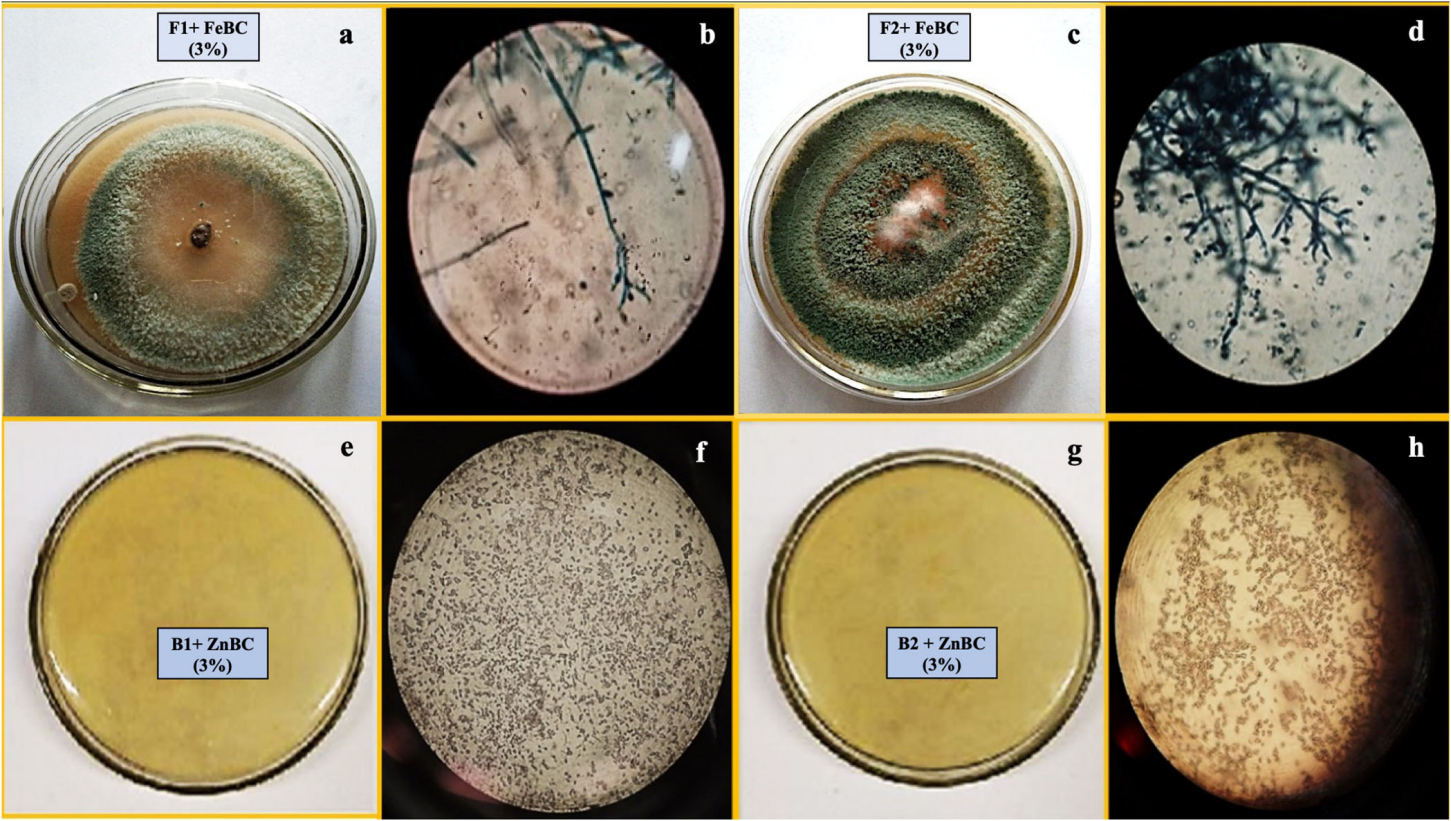
Images of *T. harzianum* fungal sp. from harvested soil **(a)** growth pattern **(b)** microscopic study and *T. viride* soil inoculated plate **(c)** growth pattern **(d)** microscopic study; images of *P. fluorescence* bacterial sp. from harvested soil **(e)** growth pattern **(f)** microscopic study and *B. subtilis* sp. from harvested soil **(g)** growth pattern **(h)** microscopic study.

Microbial spores inoculated harvested soil analyses revealed the presence of similar fungal (F1, F2) and bacterial isolates (B1, B2) as discussed for pure cultures sp. (Table 2). The following figure (Figure 3) presented the morphological study of microbial sp. isolates from inoculated soil.

**Figure 4:**
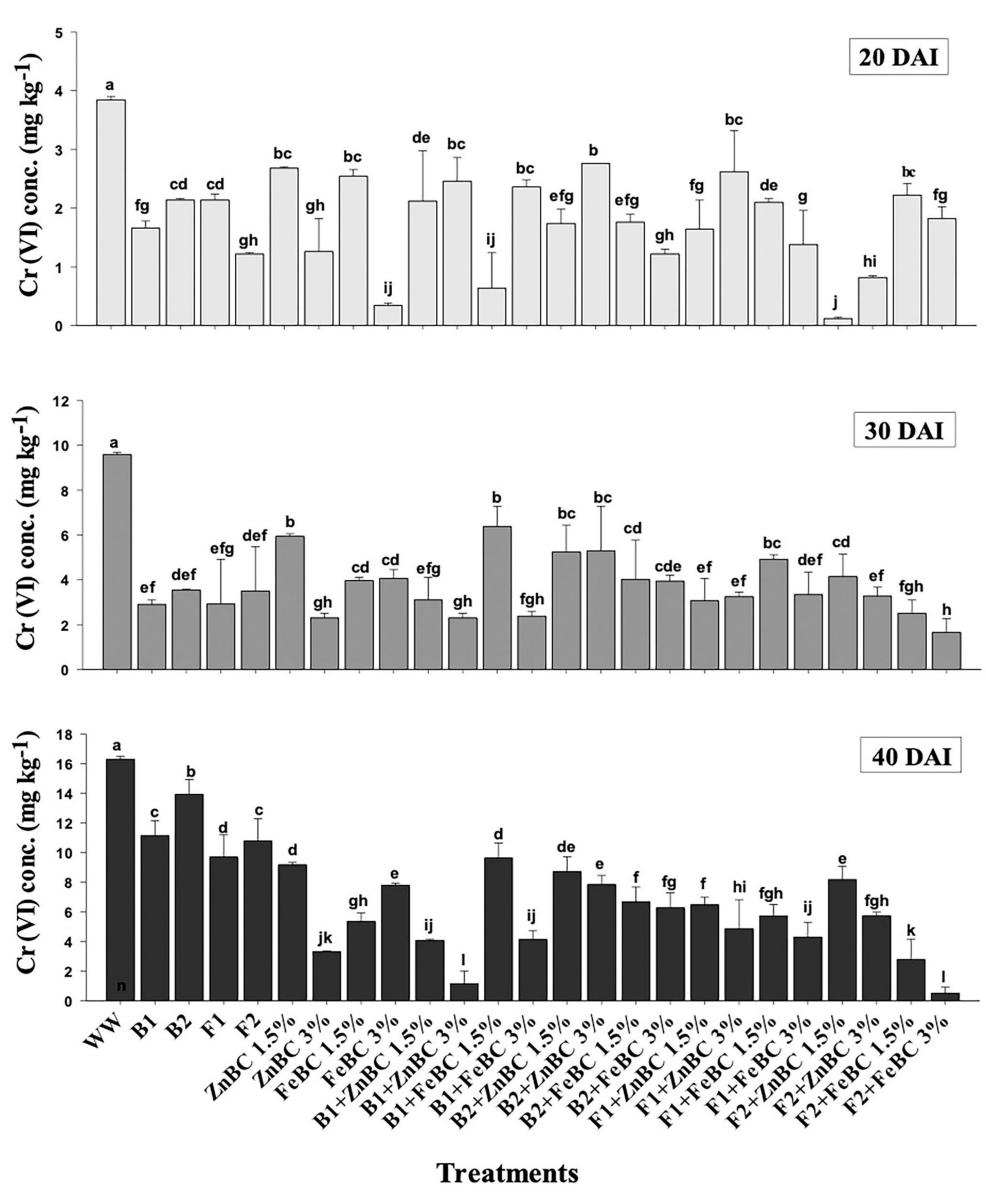
DTPA-extractable Cr(VI) concentration (Mean ± S.D) for ZnBC (1.5, 3%) and FeBC (1.5 and 3%) treatments at 20, 30, and 40 DAI periodic assessments.

There were significant (*p* < 0.05) main and first-order interactions of fungi, bacteria, ZnBC, and FeBC treatments under Cr(VI) stress as shown in Figure 4. Soil post-harvest analyses revealed an increasing rate of DTPA-extractable Cr(VI) concentration of 92%, over time (Figure 4). In WW, the extracted DTPA-Cr(VI) was 2-, 5-, and 7-folds at 20, 30, and 40 DAI, respectively (Figure 4, Table 3). However, the fungal (F1, F2) and bacterial (B1, B2) species as single soil amendments brought significant (*p* < 0.05) metal immobilization *viz*. 31.65% by B1, 14.60% by B2, 40.49% by F1, and 33.86% by F2 at 40 DAI compared to WW (Table 3). Similarly, the BC-mediated soil Cr(VI) retention trend increased with time such as 40 >30 >20 DAI compared to WW (Figure 4). It was observed that B1 + ZnBC 3% brought 92.81% metal immobilization (at 40 DAI), which was the highest among bacterial-BC co-applied treatments (Table 3). Likewise, there was a significant increase of 96.8% in Cr adsorption rate by F2 + FeBC 3% amendment relative to WW at 40 DAI (Figure 4, Table 3).

**Table 3:** Percentage (%) change in metal adsorption (R = 3 mean) after 20, 30, and 40 days of soil metal-spiking.

**Treatments**	**Cr (VI) mg kg** ^**−1**^					
	**20 DAI Mean ±S.D**	**Percentage change in Cr(VI) adsorption**	**30 DAI Mean ±S.D**	**Percentage change in Cr (VI) adsorption**	**40 DAI Mean ±S.D**	**Percentage change in Cr(VI) adsorption**
Soil	0.02 ± 0.001	–	0.021 ± 0.01	–	0.0243 ± 0.01	–
WW	3.84 ± 0.06	+191%	9.58 ± 0.09	+455%	16.3 ± 0.18	+670%
B1	1.66 ± 0.12	−56.77%	2.9 ± 0.2	−69.72%	11.14 ± 1	−31.65%
B2	2.14 ± 0.02	−44.27%	3.54 ± 0.04	−63.04%	13.92 ± 1	−14.60%
F1	2.14 ± 0.1	−44.27%	2.92 ± 2	−69.51%	9.7 ± 1.5	−40.49%
F2	1.22 ± 0.02	−68.22%	3.5 ± 1.98	−63.46%	10.78 ± 1.5	−33.86%
ZnBC 1.5%	2.68 ± 0.02	−30.20%	5.94 ± 0.12	−37.99%	9.18 ± 0.16	−43.6%
ZnBC 3%	1.26 ± 0.56	−67.18%	2.3 ± 0.2	−75.99%	3.32 ± 0.04	−79.63%
FeBC 1.5%	2.54 ± 0.12	−33.85%	3.96 ± 0.14	−58.66%	5.36 ± 0.58	−67.11%
FeBC 3%	0.34 ± 0.04	−91.14%	4.06 ± 0.4	−57.62%	7.8 ± 0.12	−52.14%
B1 + ZnBC 1.5%	2.12 ± 0.86	−44.79%	3.1 ± 1	−67.64%	4.08 ± 0.09	−74.96%
B1 + ZnBC 3%	2.46 ± 0.4	−35.93%	2.3 ± 0.2	−75.99%	1.16 ± 0.86	**−92.81%**
B1 + FeBC 1.5%	0.64 ± 0.6	−83.33%	6 ± 0.9	−37.36%	9.64 ± 1	−40.85%
B1 + FeBC 3%	2.36 ± 0.12	−38.54%	2.38 ± 0.2	−75.15%	4.14 ± 0.6	−74.601%
B2 + ZnBC 1.5%	1.74 ± 0.24	−54.68%	5.24 ± 1.2	−45.30%	8.72 ± 1	−46.50%
B2 + ZnBC 3%	2.76 ± 0.26	−28.12%	5.3 ± 1.98	−44.67%	7.84 ± 0.6	−51.98%
B2 + FeBC 1.5%	1.76 ± 0.14	−54.16%	4.02 ± 1.76	−58.03%	6.68 ± 1	−59%
B2 + FeBC 3%	1.22 ± 0.08	−68.22%	3.94 ± 0.26	−58.87%	6.28 ± 1	−61.47%
F1 + ZnBC 1.5%	1.64 ± 0.5	−57.29%	3.06 ± 1	−68.05%	6.48 ± 0.5	−60.24%
F1 + ZnBC 3%	2.62 ± 0.7	−31.77%	3.24 ± 0.2	−66.17%	4.86 ± 1.9	−70.18%
F1 + FeBC 1.5%	2.1 ± 0.06	−45.31%	4.92 ± 0.2	−48.64%	5.72 ± 0.78	−64.90%
F1 + FeBC 3%	1.38 ± 0.58	−64.06%	3.34 ± 1	−65.13%	4.3 ± 1	−73.61%
F2 + ZnBC 1.5%	0.12 ± 0.02	−96.87%	4.14 ± 1	−56.78%	8.18 ± 0.88	−49.81%
F2 + ZnBC 3%	0.82 ± 0.03	−78.64%	3.28 ± 0.39	−65.76%	5.74 ± 0.24	−64.78%
F2 + FeBC 1.5%	2.22 ± 0.2	−42.18%	2.5 ± 0.6	−73.90%	2.78 ± 1.38	−82.94%
F2 + FeBC 3%	1.82 ± 0.2	−52.60%	1.66 ± 0.6	−82.67%	0.52 ± 0.4	**−96.8%**

All the treatments have been compared with wastewater (WW) while WW has been compared with soil Cr (VI) content.

The values remained bold to distinguish them from the rest of the treatments. DAI, Day after incubation; SD, Standard deviation.

Langmuir and Freundlich adsorption isotherms were individually applied on ZnBC (1.5 and 3%) and FeBC (1.5 and 3%) against B1, B2, F1, F2 single treatments at 20, 30, and 40 DAI (Table 4). The results demonstrated that the data were highly supported by the Langmuir isotherm adsorption model based on high *R*^2^ (*R*^2^ = 0.9–1) values (Table 4). Maximum Cr(VI) adsorption capacity (41 mg g^−1^) was reported with FeBC 3% followed by ZnBC 3% (37.7 mg g^−1^), which confirmed that 3% input rate enhanced the treatments effectiveness over 1.5% input rate (Table 4).

**Table 4:** FeBC and ZnBC metal adsorption capacity (20, 30, and 40 days) determined by Langmuir and Freundlich isotherms.

**Langmuir adsorption isotherm**					**Freundlich adsorption isotherm**			
	**Q (max) or Xm (mg g** ^−1^ **)**	**k (** ***l*** **)**	**R (** ***l*** **)**	***R** ^2^*		**Qe (mg g** ^−1^ **)**	**1/*****n*** = **m (slope)**	***R** ^2^*
WW (20)	1	0.547	0.11	0.98	WW (20)	15.84	0.9	0.97
WW (30)	14	0.05	0.005	1	WW (30)	5.370	0.86	1
WW (40)	4.1	0.36	0.02	0.995	WW (40)	2.51	0.4	0.9995
ZnBC 1.5% (20)	1.06	0.77	0.157	0.9013	ZnBC 1.5% (20)	3.16	0.292	0.7782
ZnBC 1.5% (30)	9.09	0.704	0.006	0.9778	ZnBC 1.5% (30)	5.52701	0.84	0.9765
ZnBC 1.5% (40)	27.7	0.023	0.001	0.983	ZnBC 1.5% (40)	7.6	0.67	0.9696
ZnBC 3% (20)	0.672	0.066	0.01	0.9946	ZnBC 3% (20)	4.07	0.843	0.9616
ZnBC 3% (30)	11	0.067	0.005	0.9824	ZnBC 3% (30)	15.84	0.740	0.9595
ZnBC 3% (40)	38.31	0.01	0.001	0.9714	ZnBC 3% (40)	19.95	0.2	0.8632
FeBC 1.5% (20)	1.09	0.636	0.131	0.8309	FeBC 1.5% (20)	3.845	0.584	0.9202
FeBC 1.5% (30)	7.87	0.07	0.007	0.9348	FeBC 1.5% (30)	13.983	0.82	0.9147
FeBC 1.5% (40)	27.47	0.023	0.001	0.9564	FeBC 1.5% (40)	17.93	0.539	0.8853
FeBC 3% (20)	1.408	0.673	0.139	0.8631	FeBC 3% (20)	3.73	0.388	0.8268
FeBC 3% (30)	15.03	0.054	0.004	0.9931	FeBC 3% (30)	14.79	0.406	0.9756
FeBC 3% (40)	41.6	0.016	0.0009	0.9673	FeBC 3% (40)	21.35	0.198	0.8167

**Figure 5:**
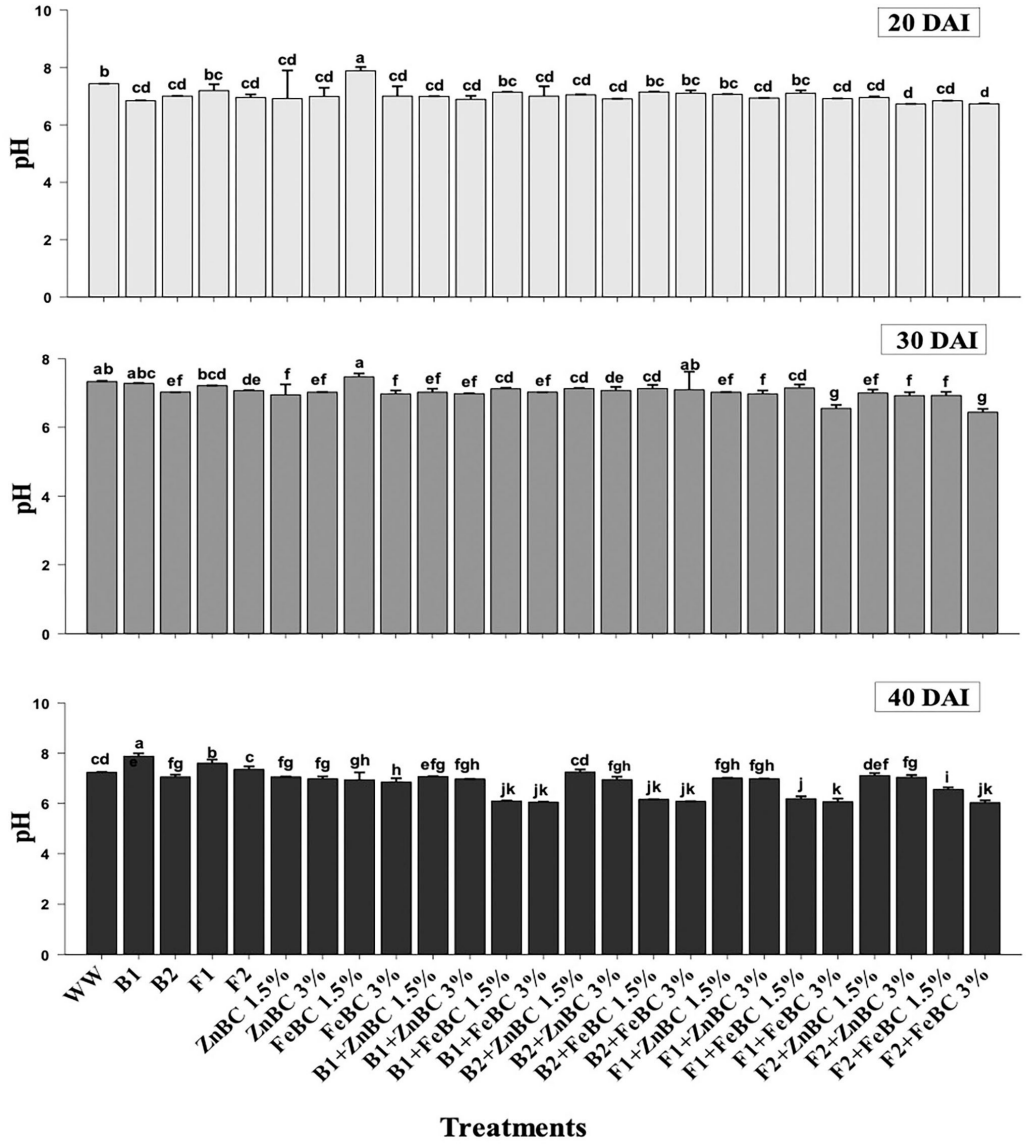
Comparative analyses of pH for treatments (Mean ± S.D) at 20, 30, and 40 DAI periodic assessments.

Data revealed significant (*p* < 0.05) variations in soil pH for main and first-order interaction of fungi, bacteria, ZnBC, and FeBC treatments under Cr(VI) stress from 20 to 40 DAI (Figure 5). The main effects showed enhancement in pH of the soil; particularly, the highest (10.65%) pH increase was observed for B1 relative to WW at 40 DAI. Additionally, all the fungal treatments except F1 + FeBC 1.5% and F2 + FeBC 1.5% caused 16.46% decrease in soil pH value relative to WW at 40 DAI.

**Figure 6:**
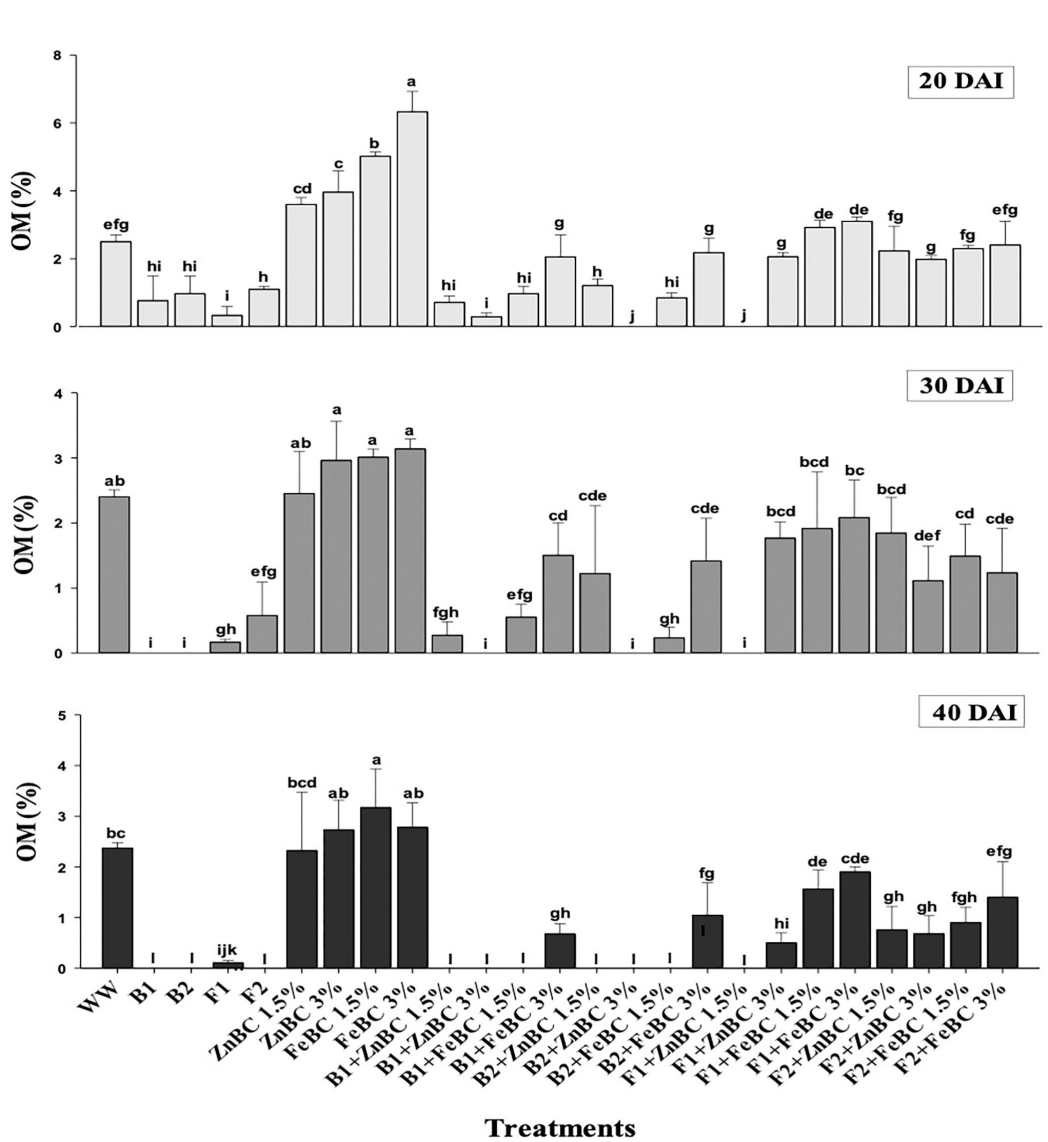
Comparative analyses of OM% content for treatments (Mean ± S.D) at 20, 30, and 40 DAI periodic assessments.

The OM content was initially (20 DAI) reported by all the treatments except B2 + ZnBC 3% and F1 + ZnBC 1.5%. The bacterial activity significantly (*p* < 0.05) degraded the OM content with the passage of time, and no OM content was reported eventually among all bacterial treatments at 40 DAI, except B1 + FeBC3% and B2 + FeBC 3% (Figure 6). Similarly, the persistent decrease in OM content was reported in those treatments where both fungal species were applied with Fe and Zn BC. There was a (37.5%) decrease in OM content estimated with F2 + FeBC 3% application, whereas a maximum increase (20%) in OM content with FeBC 1.5% was observed compared to WW at 40 DAI.

**Figure 7:**
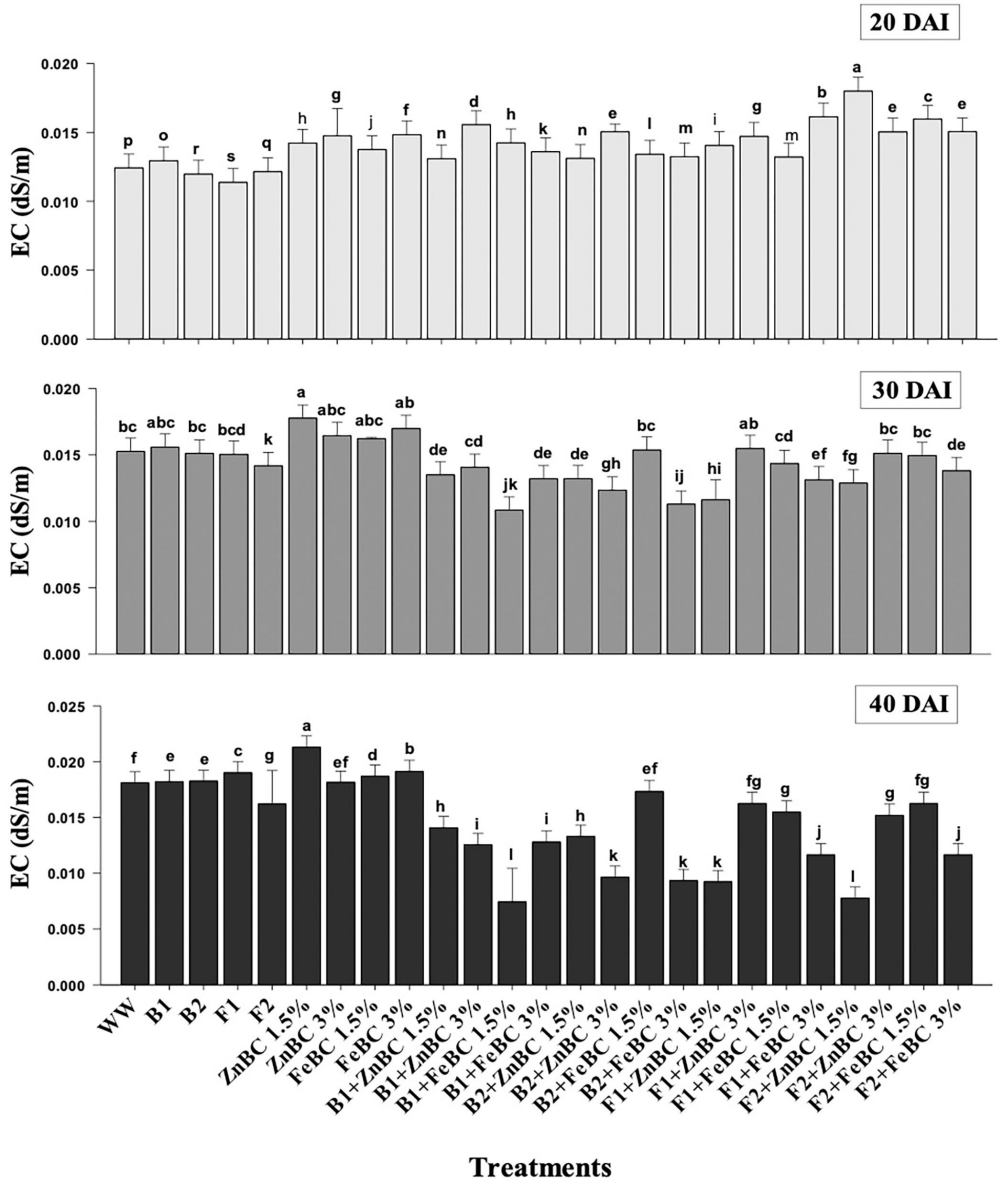
Comparative analyses of EC (dS/m) for treatments (Mean ± S.D) at 20, 30, and 40 DAI periodic assessments.

There was an increase in EC observed among first-order interaction of all the treatments at 20 DAI relative to WW; however, a significant (*p* < 0.05) reduction in EC values was obtained at 40 DAI (Figure 7). The maximum (19.4%) increase in EC value turned out to be with ZnBC1.5% treatment relative to WW, at 40 DAI. However, FeBC1.5% interaction with B1 resulted in the highest EC reduction (55.6%) among all the treatments relative to WW at 40 DAI (Figure 7).

**Figure 8:**
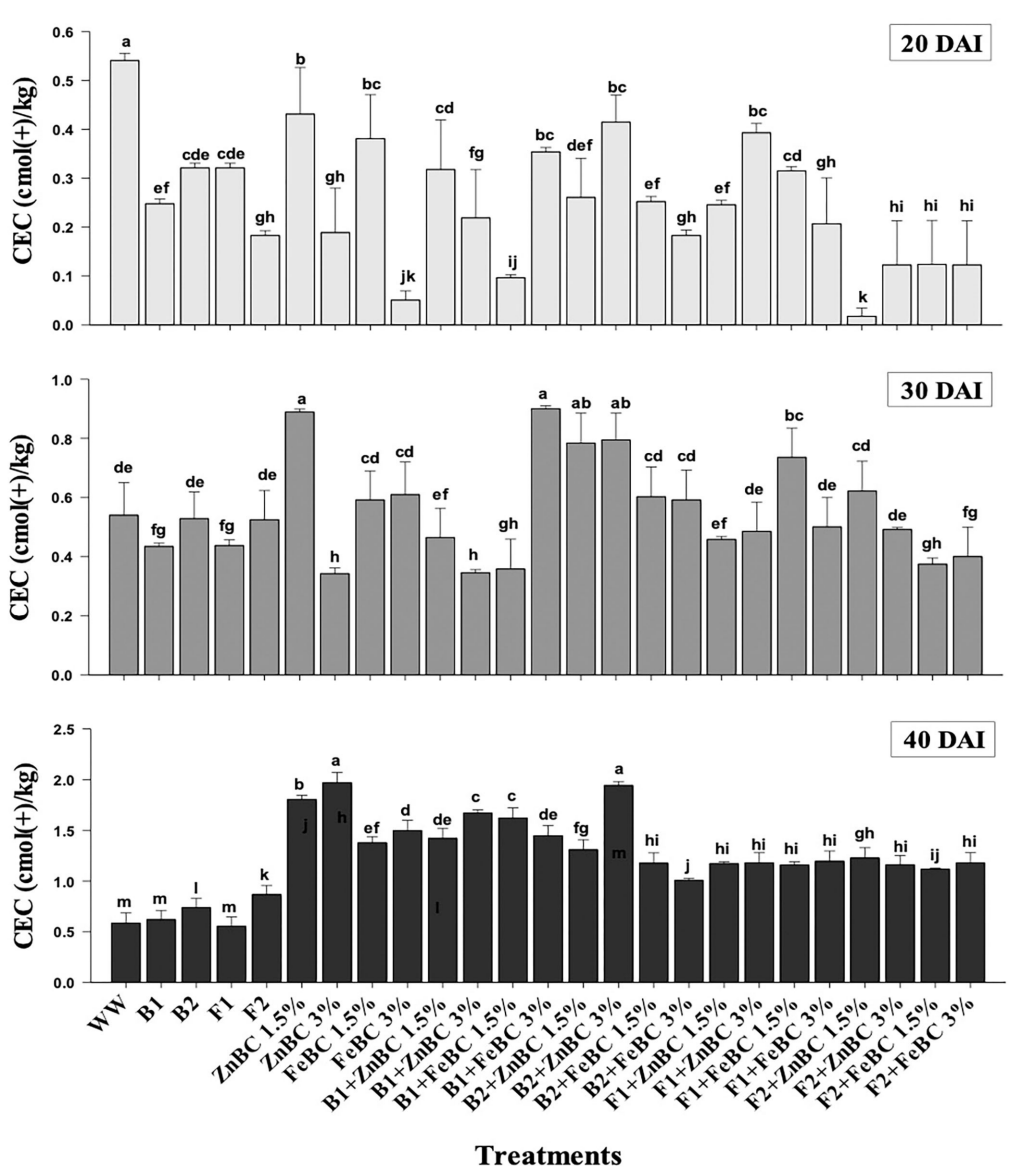
Comparative analyses of CEC [cmol (+)/kg] for treatments (Mean ± S.D) at 20, 30, and 40 DAI assessment periods.

Soil CEC of first-order interaction treatments significantly (*p*< *0.05*) increased relative to WW, from 20 to 40 DAI. ZnBC 3% as alone and along with B2 treatment caused the highest soil CEC (2-folds) relative to WW at 40 DAI (Figure 8). However, the B2 with FeBC 3% rate contributed only 1-fold (least among first-order interaction treatments) CEC rise in treated soil. It was observed that all fungal sp. applied soil samples showed lower CEC compared to bacterial appended first-order treatments relative to WW, at 40 DAI (Figure 8).

**Figure 9:**
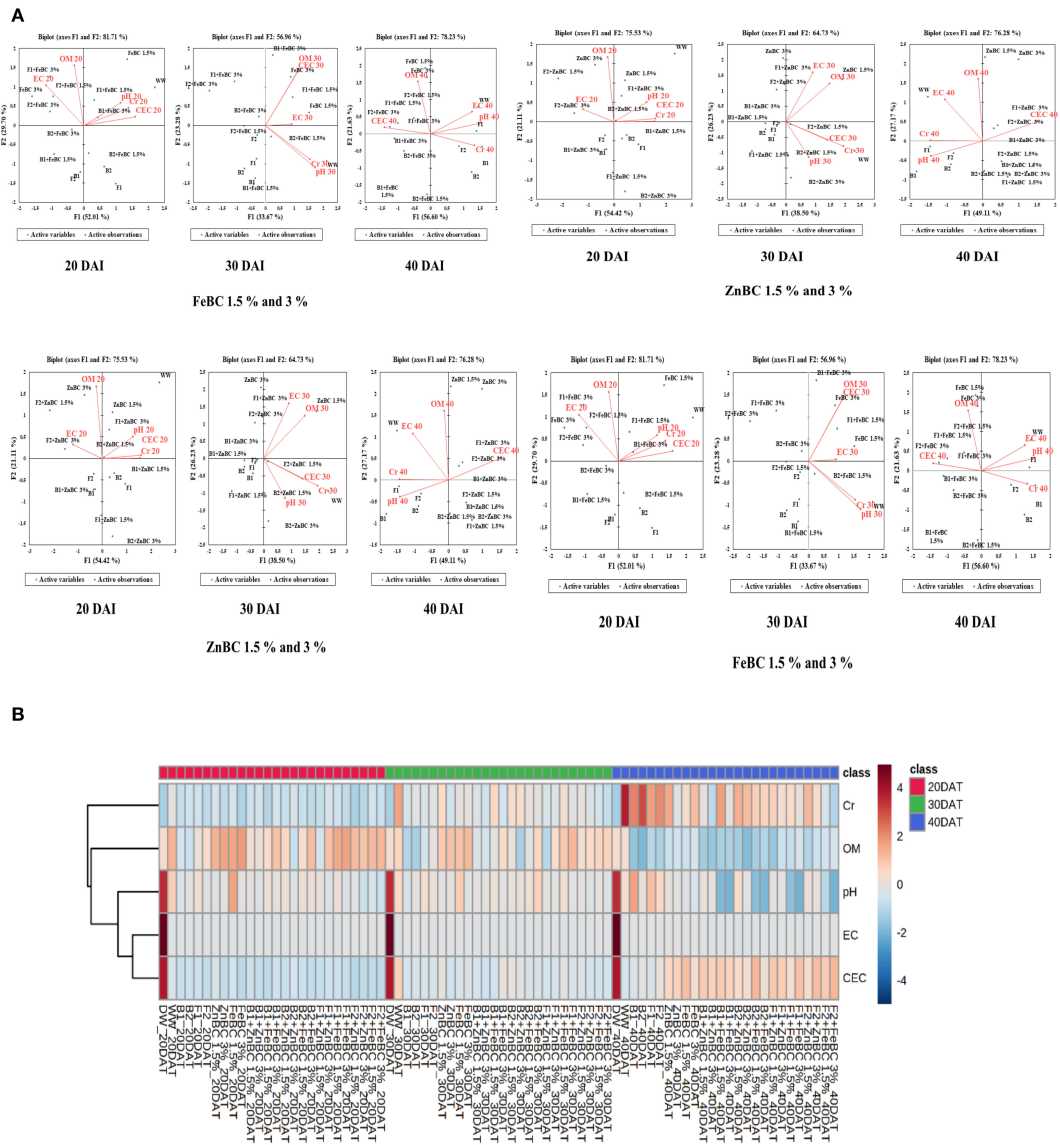
**(A)** Principal component analysis (PCA) for FeBC and ZnBC presenting data distribution co-linearity and variables co-association. **(B)** Heat map showing increasing distinction among variable values over time period of 40 days.

Principal component analyses segregated the ZnBC-bacterial while FeBC-fungal combinations as effective Cr(VI) immobilizers with >70% data variance at 40 DAI (Figure 9). ZnBC and FeBC treatments variability under Cr(VI), pH, OM, EC, and CEC parameters were visible across 20(*F* > 75%), 30(*F* > 56%), and 40(*F* > 76%) DAI (Figure 9). Similarly, the analyzed parameters (PCA active variables) also depicted the shifting trend by occupying the biplot position relative to significantly influenced treatments (active observations). Heat map data analyses visually presented a gradual increase in variability of treatments and parameters. The contrasting colors in the map depicted the variability, while less distinguishable colors showed homogeneity in variables/treatments (Figure 9B). Analyses of ZnBC and FeBC treatments through PCA biplot demonstrated that treatment-added soil Cr(VI) adsorption was increasing gradually; furthermore, the OM, EC, and pH values showed a reduction after 40 DAI. However, the treatments-applied soil CEC value increment was evident by 76.28% data variance (Figure 9).

## Discussion

### Soil Cr(VI) adsorption was time- and rate-dependent

The soil Cr(VI) adsorption followed a decreasing trend as 40 DAI > 30 DAI > 20 DAI in WW (Table 3). The significant (*p* < 0.05) metal adsorption and gradual Cr(VI) immobilization were attained by F2 + FeBC 3% (96.8%) and B1 + ZnBC 3% (92.81%) compared to WW at 40 DAI (Figure 4, Table 3). Different scientists mentioned the maximum Cr adsorption at high rates of enriched biochar, such as Liu et al. (2019a) reported that an 8% rate of Fe-enriched rice husk-BC removed 99% Cr(VI) within 2 h from soil containing 795 mg kg^−1^ Cr(VI) concentration. In another study, sheep manure Fe-enriched BC 5% w/w application to soil [100 mg kg^−1^ Cr(VI)] remediated 55% Cr concentration relative to WW within 30 days (Mandal et al., 2017). Similarly, Li et al. (2018) reported that 0.04 g Zn-enriched carbonaceous BC enhanced 91.2% metal removal from Cr(VI) solution (50–100 ppm) relative to WW within 24 h. The previous studies reported the doped BC input rate as >3% (Liu et al., 2019a, 2020); similarly, in our study, 3% BC rates with microbial application remarkably enhanced the Cr(VI) immobilization in WW-affected soil relative to all other treatments (Figure 4, Table 3). Microbes cannot break heavy metals; instead, they bring metal immobilization through surface binding and enzymatic reactions (Wang et al., 2017a). It was examined that F2 exhibited high metal sorption compared to bacterial isolates (Figure 4); thus, they remained potential candidates for hexavalent Cr decontamination by enhancing the chelation (Gan et al., 2015) and accumulation mechanisms (Zhang et al., 2020; Yu et al., 2021). Microbial function as symbionts with metals solubilization potential supports their usage in agronomic fields and for contaminated soils rehabilitation (Alam et al., 2019).

### Adsorbents Cr(VI) retention potential (isotherms analyses)

Chromium [Cr(VI)] adsorption rate by ZnBC and FeBC (1.5 and 3%) with their microbial mixes at 20, 30, and 40 DAI was checked by kinetic isotherm models (Table 4). Langmuir isotherm was recommended as the best-fitted model as most of their *R*^2^-values remained ~1. Similarly, FeBC 3% had maximum Cr(VI) adsorption capacity (Qmax) 41.6 mg g^−1^ followed by ZnBC 3% (38.31 mg g^−1^) at 40 DAI (Table 4). Basically, Langmuir isotherm suggested a monolayer Cr(VI) adsorption pattern with functional groups (COOH-, OH-) and minerals (Fe/Zn) over BC surface in soil solution (Babu and Gupta, 2008; Manzoor et al., 2013; Dad et al., 2020). Likewise, it is supposed that ZnBC and FeBC 3% dose rate contributed an increase in the number of functional groups and minerals (Fe/Zn) magnetism played a crucial role in enhanced soil Cr(VI) retention (Table 4). In a study, the Cr(VI) adsorption capacity of Zedarach (Chinaberry) wood Magnetic BC was recorded as 25.27 mg g^−1^ by Zhang et al. (2018b). Zhu et al. (2018) also applied adsorption isotherms and mentioned the Langmuir isotherm as the best-fitted model determining the Cr(VI) adsorption potential of 48.82 mg g^−1^ by Fe-enriched wetland reed BC.

### Variation in soil physicochemical parameters (pH and OM) with Cr(VI) stress under bacterial/fungal and Zn/Fe BC amendments

The experimental soil pH was 7.8 that reduced (7.4%) with the WW application at 20 DAI. Virtually, the heterogeneity of pH values increased across the treatments after 40 DAI (Figure 5). The FeBC original pH (3.67) had an acidic effect on soil pH as FeBC applied soil samples showed a pH range of 6.01–6.9. Different treatments such as F1 + FeBC 1.5% and F2 + FeBC 1.5% caused a similar reduction (16.46%) in soil pH compared to WW at 40 DAI (Figure 5). The reason for this reduction trend might be the oxidation potential of FeBC, which enhanced the exposure of Fe and acidic (carbonyl, carboxyl) functional groups, under aerobic conditions (Abrishamkesh et al., 2015). According to Kumar et al. (2007), ${\text{HCrO}}_{4}^{-}$ ions are readily adsorbed over the soil surface by electrostatic attraction under acidic conditions, as the probability of H^+^ ions exchange increases with negatively charged ions. However, ZnBC (pH 4.7)-added soil samples exhibited a 6.9–7.25 pH range (Figure 5). The Cr(VI) translocation and mobilization majorly depend on its oxidative state and medium pH (Attah and Melkamu, 2013). Furthermore, acidic to neutral pH might induce different metal immobilization mechanisms such as reduction, ion exchange, precipitation, and metabolic metal transformations (Palansooriya et al., 2020). The highly effective F2 + FeBC3% and B1 + ZnBC3% treatments had pH values of 6.03 and 6.97, respectively. Thus, the treatments exclusively contributed in setting the medium pH which influences microbial viability, Cr(VI) oxidative state, and minerals/OM availability.

There was no OM content reported in all bacterial treatments at 40 DAI except B1 + FeBC3% and B2 + FeBC 3% (Figure 6). Organic matter oxidation, microbial metabolic activities, and metal reduction might be the reasons for OM content variation with time (Huang et al., 2017; Liu et al., 2020). Bacteria with fast metabolic activity might consume soil OM at a faster rate compared to fungi (Figure 6). However, OM was present in all fungal applied BC (ZnBC, FeBC) treatments, except F1 + ZnBC 1.5% at 40 DAI. Fungi mycelium grows slowly and retains OM, which contributes to carbon balance and metal particles retention in soil (Xu et al., 2018). Thus, FeBC-fungi inoculated soil had a high potential for metal immobilization, carbon sequestration (high organic content observed), and long-term soil minerals management. With FeBC 1.5%, the maximum increase (20%) in OM content was observed compared to WW at 40 DAI (Figure 6). The variability can be attributed to aerobic experimental conditions and subsequent relative exposure to air.

### Variation in soil physicochemical parameters (EC and CEC) with Cr(VI) stress applied under bacterial/fungal and Zn/Fe BC amendments

In this study, the soil EC ~0.8 dS/m was reported before commencing the experiment, whereas the optimal EC ranges between 1.10 and 5.70 dS/m for fertile soil (Ghorbani et al., 2019). The WW application significantly (*p* < 0.05) reduced (97.73%) the soil EC as compared to the natural soil (Figure 7). However, the EC value of the soil increased for the main effects of all treatments as we moved from 20 to 40 DAI, while it remained unchanged or lower for the combined BC and microbial treatments over time (20–40 DAI) (Figure 7). Few research studies already reported a rise in soil EC with time as enriched BC application might acquire high reaction kinetics (Masulili et al., 2010; Ghorbani and Amirahmadi, 2018). Contrarily, few studies mentioned non-significant changes in EC value of soil even after the addition of organic amendments (Jien and Wang, 2013; Mavi et al., 2018). The EC of F2 + FeBC 3% and B1 + ZnBC 3% treatments was reduced by 27.1 and 33.3%, relative to WW at 40 DAI, respectively (Figure 7). Low EC represents the less availability of nutrients, while high EC value indicated the presence of excessive nutrients (Liu et al., 2020). Microbial metabolic activities and carbon/ nitrogen turnover directly influence the nutrients balance of soil (Muniz et al., 2014; Chen et al., 2017, 2021). Varied and low EC value range (relative to WW) for all ZnBC/FeBC microbial combinations might be due to an increase in metal-salt-OH groups or ionic and electrostatic interactions (Saffari et al., 2016; Liu et al., 2020). According to PCA, the EC value was significantly (*F* = 76.28% for ZnBC; *F* = 78.23% for FeBC) and positively co-related to DTPA-extractable Cr(VI) content of soil (Figure 9).

The optimal soil CEC has a range of ~4.5–5 cmol(+)/kg for sandy loam soil (Olorunfemi et al., 2016). The WW application significantly (*p* < 0.05) reduced (86.5%) the soil CEC value (Figure 8). This reduction might be attributed to Cr(VI) adsorption mechanism at the initial days (20 DAI) (Liu et al., 2020); however, microbial application with BC (first-order interaction) showed a gradual increment in soil CEC. After 40 DAI, there was a 2-fold increment in the CEC value observed for B2 + ZnBC 3% relative to WW (Figure 8). Different scientists have proposed different reasons such as it could be due to oxidation of BC functional groups, increased mobility of Cr(VI) due to microbial activity, competition between OH^−^ groups and metal anions and disassociation of mineral and metal components (Wang et al., 2017a,b; Liu et al., 2020; Yu et al., 2021). Furthermore, alterations in H^+^ ions balance, metal transformation (precipitation), DOC bioavailability, microbial mobility, and metabolic activity rate (polyphenol oxidase, dehydrogenase, catalase) in soil matrix might be other influential factors for CEC variation (Bandara et al., 2017). Biochar (BC) was approved to be an effective soil conditioner that extracted Cr from the soil solution and thus subsequently improved the soil CEC (Figure 8). Jien and Wang (2013) also reported an increment in the soil CEC value by application of BC having high porosity and large surface area. The sandy loam contains low mineral contents due to lesser silt and clay fractions; thus, BC acquires a high surface charge in sandy loam soil compared to clay soil (Aljoumani et al., 2017). Similarly, the CEC percentage increase was higher for sandy loam than clay, as reported by Ghorbani et al. (2019). Increasing CEC indicates soil nutrients holding capacity, which ultimately depends on soil biota, adsorbents, moisture content, and texture (Hao et al., 2014). CEC and OM values for tested soil samples depicted the positive co-relations that pointed out OM as a reactive material affecting the soil Cr(VI) adsorption kinetics and CEC (Figure 9). PCA segregated ZnBC/FeBC-fungal assemblages applied soil samples with high CEC and OM values. The above-mentioned factors might be the reason for significantly (*p* < 0.05) enhanced metal immobilization (96.2%) by F2 + FeBC3% treatment applied to the soil which had high CEC (85.7%) and low pH value (11.7%) relative to WW and viable OM content at 40 DAI (Figures 9A,B).

## Conclusion

Both fungal sp. with enriched BC (Zn/Fe) proved to be an excellent combination for soil Cr immobilization and showed slow OM oxidation relative to bacterial sp. Thus, FeBC3% + F2 application remained the best treatment for Cr(VI) immobilization as 96.8% soil Cr(VI) adsorption was achieved relative to WW. Similarly, soil Cr(VI) adsorption increased to 92.81% with ZnBC 3%+ B1 relative to WW. Langmuir adsorption isotherm gave FeBC 3% group treatments maximum Cr(VI) adsorption capacity (Qmax) 41.6 mg g^−1^ followed by ZnBC 3% group treatments as 38.31 mg g^−1^ at 40 DAI. Soil pH turned slightly acidic with FeBC and remained neutral with ZnBC promoting the Cr(VI) reduction and speciation processes. We concluded that combined application gave better metal remediation outcomes relative to amendments single application. Biochar was enriched with Fe and Zn minerals because these minerals are deficient in soils of Pakistan and also have nutritional value. This is the first study to report the use of zinc-enriched BC with microbial cultures. Similarly, limited studies were found on Fe-enriched BC co-application with microbial amendments. Mostly, the literature was available on enriched BC and microbial individual or sole applications. Under the comparative analyses' framework, we tried to find out how the combined application was beneficial for soil health and what alterations are occurred in physicochemical properties.

Eventually, this study implies that microbes along with Zn/Fe doped BC resulted in low pH, high OM, and CEC, which ultimately played a role in maximum Cr(VI) adsorption from WW applied soil. However, study can further be extended to determine the soil physicochemical characteristics optimization to promote soil structural stability and high crop productivity with biochar-microbial application. Additionally, plant growth variables can be assessed under the set of these remedial approaches.

## Data availability statement

The original contributions presented in the study are included in the article/Supplementary material, further inquiries can be directed to the corresponding author/s.

## Author contributions

W-u-DK conceived the idea of this research paper. MB, W-u-DK, MA, and FN designed the methodology of this research paper. MB conducted this research with the help of W-u-DK, MA, MH, and MNA. SR, ZD, FN, and MNA revised and critically reviewed the manuscript. All authors contributed to the subsequent development and approved the final manuscript.

## Funding

The authors acknowledge the financial support provided by the National Key R&D program of China (2021YFD1700900), Central Public interest Scientific Institution Basal Research Fund (Y2022GH10), and the Agricultural Science and Technology Innovation Program of the Chinese Academy of Agricultural Sciences (Grant No. CAAS-ASTIP202101).

## Conflict of interest

The authors declare that the research was conducted in the absence of any commercial or financial relationships that could be construed as a potential conflict of interest. The reviewer MM declared a shared affiliation with the author MH to the handling editor at the time of review.

## Publisher's note

All claims expressed in this article are solely those of the authors and do not necessarily represent those of their affiliated organizations, or those of the publisher, the editors and the reviewers. Any product that may be evaluated in this article, or claim that may be made by its manufacturer, is not guaranteed or endorsed by the publisher.

## Supplementary material

Supplementary Figure 1 Pure cultures fungal (F1: 1; F2: 2) DNA bands appeared by gel electrophoresis **(a)** before PCR amplification **(b)** after PCR amplification (1 denotes to T.harzianum while 2 denotes to T.viride species; the alphabets show sample replicates); Pure cultures bacteria (B1: 1; B2: 2) DNA bands appeared by gel electrophoresis **(c)** before PCR amplification **(d)** after gel electrophoresis (1 denotes to P. fluorscence while 2 denotes to B. subtilis; the alphabets show sample replicates).

Supplementary Figure 2 Changes in soil physicochemical properties under organic and microbial amendments over time period of 40 days.
