# Supplementary figures and images for: Microbial-assisted soil chromium immobilization through zinc and iron-enriched rice husk biochar
Source: Front Microbiol. 2022 Sep 12;13:990329. doi: 10.3389/fmicb.2022.990329 (PMC9511223; doi:10.3389/fmicb.2022.990329)

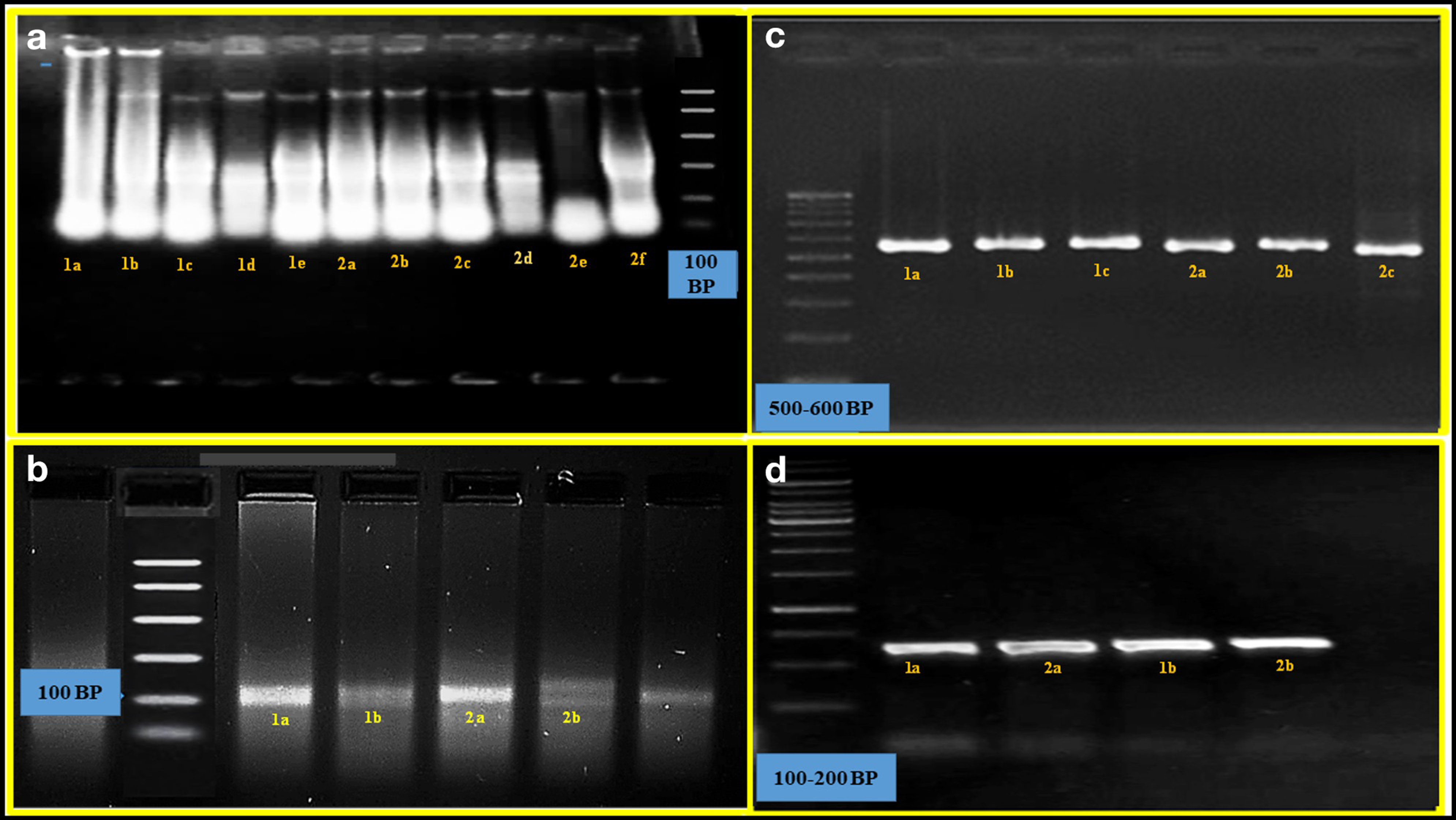

Supplement: Supplementary Figure 1 — Pure cultures fungal (F1: 1; F2: 2) DNA bands appeared by gel electrophoresis (a) before PCR amplification (b) after PCR amplification (1 denotes to T.harzianum while 2 denotes to T.viride species; the alphabets show sample replicates); Pure cultures bacteria (B1: 1; B2: 2) DNA bands appeared by gel electrophoresis (c) before PCR amplification (d) after gel electrophoresis (1 denotes to P. fluorscence while 2 denotes to B. subtilis; the alphabets show sample replicates). [file Image_1.jpg]

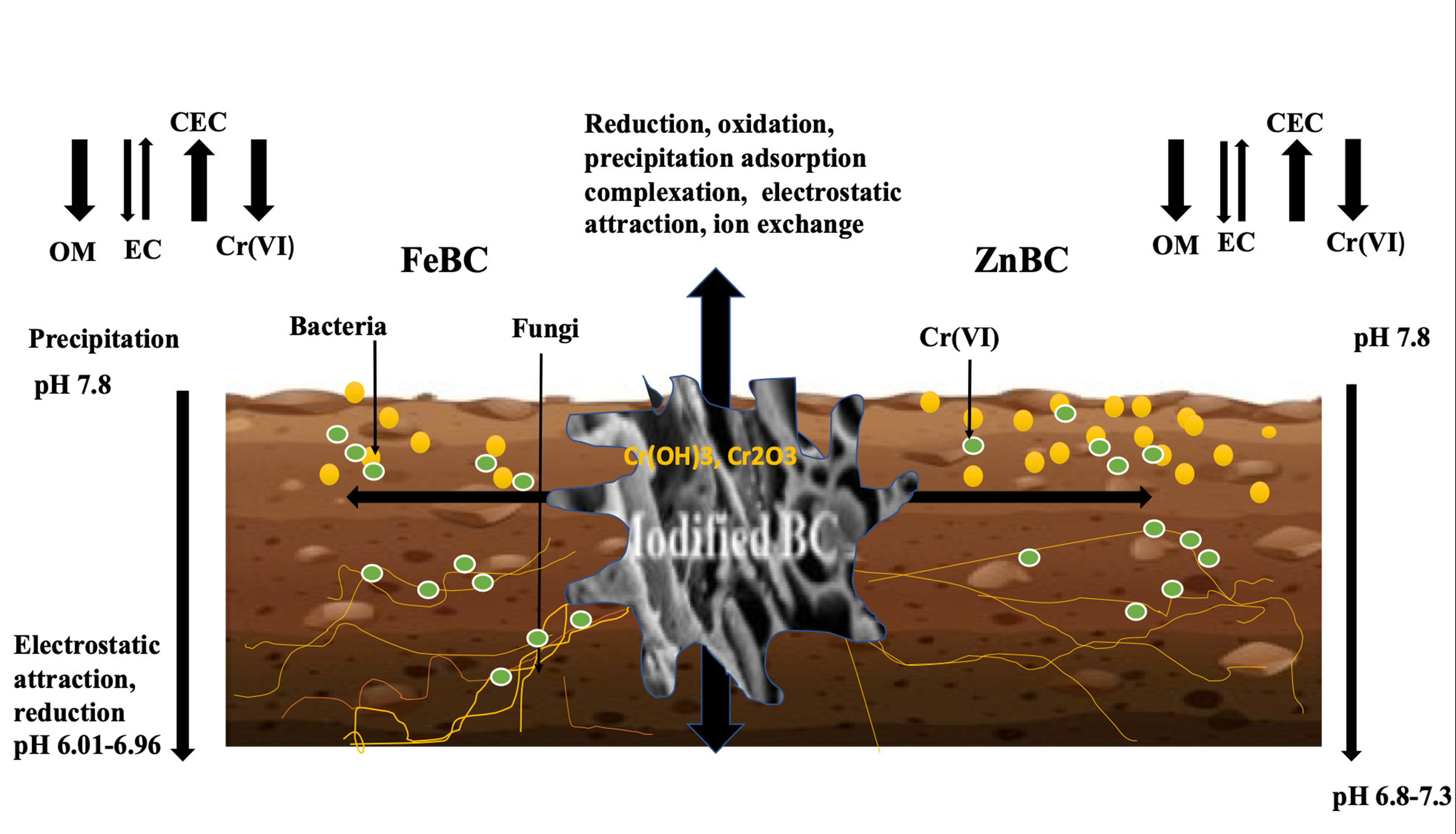

Supplement: Supplementary Figure 2 — Changes in soil physicochemical properties under organic and microbial amendments over time period of 40 days. [file Image_2.JPEG]
